# Evaluation of Temporomandibular Joint by Anesthetists in Florida When Conducting Orotracheal Intubation—A Pilot Study

**DOI:** 10.3390/jcm9103229

**Published:** 2020-10-09

**Authors:** Inae C. Gadotti, Melanie Geronimo, Gabriela Rodriguez, Stephanie Caceres, Yasmine Campbell, Jorge Valdes, Derrick Glymph

**Affiliations:** 1 Department of Physical Therapy, Florida International University, Miami, FL 33199, USA; mgero003@fiu.edu (M.G.); grodr205@fiu.edu (G.R.); scace001@fiu.edu (S.C.); 2 Nurse Anesthetist Practice, Florida International University, Miami, FL 33199, USA; ycampbel@fiu.edu (Y.C.); jvalde@fiu.edu (J.V.); dglymph@fiu.edu (D.G.)

**Keywords:** temporomandibular joint, orotracheal intubation, anesthesia

## Abstract

Background: Orotracheal intubation has been considered a risk factor for the development or exacerbation of disorders related to the temporomandibular joint (TMJ). The objective of this pilot study was to evaluate TMJ assessment performed by anesthetists in Florida when conducting orotracheal intubation. Methods: An online questionnaire was created using Qualtrics. The survey included 22 questions related to demographics, TMJ related to anesthesia procedures, and education/awareness regarding TMJ related to intubation. Descriptive statistics and cross-tabulation analysis were performed. Results: One hundred and eight providers participated (mean 46 years-old ± 12; 59% female). About 50% reported to always evaluate TMJ prior non-emergency intubation. Of those, 56% used an alternative intubation technique based on the TMJ status and 60% never/rarely evaluated TMJ post-intubation. Twenty-five percent reported they did not know of possible intubation effects on TMJ and 47% reported that they did not receive adequate information/education on TMJ management related to intubation in school. After participation, 81% reported to be more aware of the importance of evaluating TMJ. Conclusions: Only half of the providers who frequently performed intubation in Florida evaluated TMJ prior to intubation. This survey increased the awareness regarding the importance of evaluating TMJ when conducting intubation. This may contribute to reducing TMJ issues associated with non-emergency intubations.

## 1. Introduction

General anesthesia with orotracheal intubation has been considered a risk factor for the development or exacerbation of temporomandibular disorders (TMD) [1]. TMD is the second most common musculoskeletal pain after low back pain. Approximately 33% of the population has at least one TMD symptom and from 3.6 to 7.0% of the population has severe TMD [2]. The incidence of TMD following intubation was reported to be 5%, and the worsening of a previous TMD was shown to be present in 44% of patients [3]. Temporomandibular joint (TMJ) injuries represent 10% (27 out of 266) of airway trauma claims with TMJ pain and dislocation being the most common complications [4,5]. Damage to the TMJ may be due to excessive forces being applied to achieve a maximum opening of the patient’s mouth in an attempt to complete an intubation or other procedures such as bronchoscopy. In addition, the loss of muscle tone due to the unconsciousness and muscular relaxation of a patient under general anesthetic can predispose to greater joint mobilization [6].

The majority of anesthesiologists focus on the TMJ function evaluation as it relates to optimal intubating conditions [7]. Difficulties with intubation are especially common in patients with limited mouth opening such as those with TMJ ankyloses [8,9,10,11]. However, proper TMJ assessment is required both before and after anesthesia in order to avoid or minimize temporomandibular complications [6,12]. TMJ evaluation should be a component of the preoperative airway physical examination as suggested by The American Society of Anesthesiologist practice guidelines [13]. According to Agro et al., [3] this will allow the anesthesia providers to choose the best intubation technique and to reduce the risk of the new onset of TMJ issues, or the worsening of misdiagnosed TMD after intubation. For instance, the retrograde nasotracheal intubation was found to be an effective and useful technique for airway control in patients with limited mouth opening [14]. However, no studies have been performed to evaluate if the anesthesia providers are actually evaluating TMJ status prior and after anesthesia with intubation, or if different intubation procedures are being selected based on the patient’s signs and symptoms, including limited mouth opening.

Even though some studies show that general anesthesia with intubation can influence the onset and worsening of TMD [6], the literature is still very limited on this topic. Moreover, in a recent longitudinal study, no difference was found in the incidence of signs and symptoms of TMD in patients who underwent orotracheal intubation compared with the patients who underwent surgery without intubation [15]. Patients were assessed before surgery, and after 1 day and 3 months of the surgery. However, the study presented some limitations: only TMD of muscular origin was evaluated, and the TMD pre-assessment was self-reported by the patients after the surgery with no measurements of mouth opening, the 3 months’ follow-up was performed by phone, and only older patients were included (mean of 66 years for study group, and 54 years for the control group). According to a systematic review of the literature, more studies of higher evidence quality need to be conducted on TMJ issues in relation to general anesthetic and sedation procedures [6].

Therefore, the objective of this pilot study developed by physical therapy and nursing faculty and students was to evaluate the temporomandibular joint assessment performed by anesthesia providers in Florida when conducting general anesthesia with orotracheal intubation. This study will provide relevant preliminary information on the evaluation of TMJ status before and after intubation by anesthesia providers, the reported frequency of TMJ issues after intubation, the procedures performed to minimized temporomandibular complications with intubation, and the quality of information received on TMJ issues with intubation during entry-level education. In addition, this study may increase the awareness of anesthesia providers regarding the importance of evaluating TMJ status before and after intubation. The increase in awareness may contribute to reducing TMJ issues with intubation and possibly increase collaborations between nurse anesthetists and other healthcare providers in the management of these patients such as physical therapists.

## 2. Materials and Methods

### 2.1. Study Design

This was a pilot cross-sectional descriptive study approved by the Institutional Research Board from Florida International University (IRB-19-0159-AM01).

### 2.2. Recruitment

An online questionnaire was sent to 5193 anesthetists in Florida. A contact list of anesthetists was obtained from the Florida Health-Care Practitioner Portal. The anesthetists were contacted through email, which included a statement of the study objectives and a link to the online survey. Prior to completing and submitting the survey, the anesthetists were informed about their consent to participate in the study and authorized the researchers to analyze their responses. The anesthetists were informed that no identifiable information would be published or released and that participation was voluntary. In addition, they were informed that they would receive no compensation for participating in the study. Four reminder emails were sent from the initial recruitment email.

### 2.3. Questionnaire

A questionnaire was created using Qualtrics^®^ online survey software (Qualtrics Labs Inc., Provo, UT, USA). The questionnaire included questions related to demographics and work experience, general anesthesia procedures, temporomandibular joint related to anesthesia procedures, and education and awareness regarding TMJ related to intubation (Appendix A). The online survey was created so that it would take no longer than 10 min to complete.

### 2.4. Data Analysis

Descriptive statistics were calculated to analyze the responses. Data were presented as the total number of participants (*n*) and frequency (%). Written information provided by participants was considered and presented. Cross tabulation analysis was also used to examine the data and discuss whether there was a correlation among intubation procedures, education and awareness regarding TMJ related to intubation using Qualtrics^®^ analysis software.

## 3. Results

### 3.1. Participants’ Demographics and Characteristics

A total of 108 anesthetists completed the survey. The mean age of the participants was 46 ± 12 years with a range of 28–72 years of age. Forty-one of the participants (41%) were male and fifty-nine of the participants (59%) were female. The majority of the participants (93%) were currently in practice and had up to 20 years of experience. Thirty-six providers stated having another healthcare license in addition to their certified registered nurse anesthetist (CRNA) license including advanced practice registered nurse (APRN) and registered nurse (RN). Table 1 shows detailed information on the participants’ demographics.

### 3.2. General Anesthesia Procedures and Temporomandibular Joint (TMJ) Related to Anesthesia Procedures

Seventy percent of the responders answered always or very often to performing general anesthesia with intubation. The majority of them (91%) considered themselves proficient regarding the performance of general anesthesia with orotracheal intubation.

Sixty-nine percent believed it is very important or important to evaluate the TMJ prior to intubation. Seventy-two percent (72%) of responders reported that they always or very often evaluated the TMJ prior to non-emergency intubation. Fifty percent reported to always evaluate TMJ prior to non-emergency intubation. Seventeen percent (17%) rarely or never evaluated TMJ status prior intubation.

Typical procedures included in the TMJ evaluation by the participants are shown in Table 2. Other procedures reported by the responders in the open-ended question were: evaluation of the dental status, tongue size, obstructive sleep apnea risk, history taken related to prior jaw surgery, and evaluation of whether patients can protrude the mandible with or without pain. Forty participants (57%) reported that they rarely or never informed patients about the possible risks of intubation to the TMJ prior to non-emergency intubation. Only 18% reported to always or very often inform patients about the risks.

Of those who reported always evaluating the TMJ prior to intubation, 56% reported that alternative techniques were implemented when the TMJ status was compromised. Alternative techniques included GlideScope, fiber optic scope, retromolar, C-Mac, nasal intubation, McGrath MAC video laryngoscope, laryngeal mask airway and video-assisted laryngoscopy. Nineteen (44%) providers reported using the Glidescope as an alternative intubation technique when the TMJ status was compromised.

Seventy-one percent (71%) of the providers reported that they rarely or never evaluated TMJ status post-intubation. Only 11% of them always or very often evaluated TMJ status post-intubation.

From the providers that evaluated TMJ status post-intubation, 76% reported that patients rarely, very rarely, or never complained about TMJ issues post-intubation. TMJ dislocations were reported to be rare or very rare by 63% of participants (Table 3). Most participants (97%) reported that post-procedures for TMJ problems after intubation are rarely or never performed. From the 3% who reported to perform post-surgery procedures after TMJ problems post-intubation, the following were performed according to the open-ended questions: “Sedation to relax the muscles of mastication to allow repositioning of the mandible”, “Oral and maxillofacial surgery consultation and assessment”, “unfreeze jaw by an ear, nose and throat specialist”, and “sedation to reduce to subluxation and jaw subluxation reduction”.

### 3.3. Education on TMJ Related to Intubation

Twenty-five percent (25%) of the anesthesia providers reported not knowing that orotracheal intubation could cause a new onset of TMJ dysfunction or the worsening of a previous TMD, while forty-five percent (45%) reported knowing this to some extent. When asked if they received training related to TMJ conditions with intubation while in school, 46% replied “no”. Thirty percent (30%) reported that they received very little, and seventeen percent (17%) reported receiving no information/education on the management of TMJ issues related to intubation while in school. Only 9% reported that they received a great extent of information/education while in school. After participating in this survey, 81% of the providers reported that they were more aware of the importance of evaluating the TMJ before and after orotracheal intubation.

## 4. Discussion

This was the first study to evaluate TMJ assessment performed by anesthetists in Florida when conducting orotracheal intubation. According to the results, most providers (72%) reported to always or very often evaluate the TMJ prior to non-emergent intubation. However, of those who reported always evaluating the TMJ prior to intubation, 60% reported never or rarely evaluating TMJ status post-intubation and only about half (56%) reported that alternative techniques were implemented when the TMJ status was compromised.

Anesthesia providers should be evaluating the TMJ pre- and post-intubation. TMJ assessment is required both before and after anesthesia in order to avoid or minimize temporomandibular complications as suggested by The American Society of Anesthesiologist practice guidelines [13]. The assessment of the TMJ serves to choose the best intubation technique in order to reduce the risk of a new onset of TMJ issues, or the worsening of pre-existing TMD. However, according to the results of this study, anesthetists are not frequently changing the intubation technique based on the TMJ status of the patient. Further studies should evaluate if alternative intubation techniques are not being utilized due to (1) the lack of further TMJ evaluation and therefore the need is not considered, (2) the focus is only on an optimal intubating condition, or (3) other techniques are not feasible and/or availabel such as the retrograde nasotracheal intubation. This technique was found to be effective for patients with limited mouth opening [14].

The majority of the anesthetists (97%) reported that procedures for TMJ problems after intubation are rarely or never being performed. This may be due to the small frequency of TMJ issues/dislocation reported after the intubation by the providers’ participants. However, the majority (71%) rarely or never evaluate TMJ status post-intubation. Therefore, temporomandibular disorders may go undiagnosed if TMJ status is not evaluated after intubation. The unawareness of the anesthesia providers with TMJ problems after intubation may be a contributing factor for delayed TMD diagnosis and its treatment [5,7].

Twenty-five percent (25%) of anesthesia providers reported not knowing that tracheal intubation could cause a new onset of TMJ dysfunction or the worsening of misdiagnosed TMJ disorders, while forty-five percent (45%) reported knowing this to some extent. The fact that 46% of participants reported not receiving any training related to TMJ conditions while in school could be correlated with the lack of awareness that orotracheal intubation has been considered a risk factor for the development or exacerbation of disorders related to the TMJ. More information about TMJ evaluation concerning general anesthesia with orotracheal intubation, the risks of the TMJ associated to intubation, and alternative techniques that can be used to minimize the risks should be included in the anesthesia programs to ensure that providers are compliant with the guidelines from the American Society of Anesthesiologist practice. 

After participating in this survey, eighty-one percent (81%) of the surveyed providers reported they were more aware of the importance of evaluating the TMJ before and after intubation. This will provide anesthetists the opportunity to use this information to increase the awareness of temporomandibular disorders related to orotracheal intubation. Providers can choose to evaluate TMJ status prior to general anesthesia with orotracheal intubation in order to use alternative techniques and decrease the risks of TMD.

### Practical and Research Implications for Physical Therapy

Physical Therapy is one of the most effective conservative treatments for TMD among others such as behavioral therapy and occlusal appliances. The most important contribution by physical therapists is the identification of the musculoskeletal components that contribute to the symptoms of the patient. Because the TMJs are part of the musculoskeletal system, physical therapists can treat TMJ issues with similar interventions as they would in most other body joints. Especially for patients with chronic TMD, physical therapists will work in collaboration with dentists, speech pathologists, physicians, and psychologists [16,17,18,19].

Because orotracheal intubation has been considered a risk factor for the development or exacerbation of TMD, more collaborations between anesthesia and physical therapy programs is also suggested. Interdisciplinary lectures between physical therapy and nurse anesthesia may be beneficial to increase awareness among students in their programs. Increased awareness can help further increase collaborations between these disciplines in the recognition and prevention of temporomandibular disorders associated with orotracheal intubation.

In addition, more research involving physical therapy and anesthesia programs to evaluate and prevent TMJ issues with intubation is needed. Ideally, physical therapists, nurse anesthetists, and anesthesiologists should be involved in the development of studies regarding the evaluation of TMJ in patients going to general anesthesia with orothracheal intubation. This is an important topic to be addressed in future rehabilitation research.

## 5. Study Limitations and Future Directions

This study presents some limitations. Due to the small sample size (low response rate) and therefore limited generalizability of the findings, the results of this study should be interpreted with caution. The authors believed that not all email addresses were updated in the list provided from the Portal used and that could have affected the amount of responses received. In addition, not all contacted anesthetists performed orotracheal intubation and that limited the participation in the survey. In order to maximize participation, the survey was made to be short (10 min to complete). This study included certified registered nurse anesthetists registered on a database from the State of Florida only. Future studies should include a larger sample, include anesthesiologists, and include providers from other states in order to increase the generalizability of the study. However, this study presents relevant preliminary information regarding the evaluation of TMJ by anesthetists regarding general anesthesia with orotracheal intubation in the State of Florida. Preliminary information is also provided regarding their level of awareness about the importance of the TMJ assessment. A protocol of the evaluation of TMJ by anesthesia providers should be developed in future studies.

## 6. Conclusions

Only half of the providers who frequently perform intubation in Florida evaluated TMJ status prior to intubation and 71% reported not evaluating TMJ post-intubation. This survey helped to increase the awareness of anesthetists regarding the importance of evaluating TMJ when conducting intubation. This awareness may contribute to reducing TMJ issues associated with non-emergency intubations.

## Figures and Tables

**Table 1:** Participants’ demographics and characteristics.

Variable	Value *
Age, years (mean, standard deviation and range)	46 ± 12 (28–72)
Gender, male/female (total number, percentage)	35 (41%)/51 (59%)
Highest level of education (total number, percentage)	
Bachelor’s Degree	9 (9%)
Clinical Master’s Degree	32 (33%)
Clinical Doctoral Degree	15 (16%)
Academic Master’s Degree	34 (35%)
Academic Doctoral Degree (PhD)	6 (6%)
Practice status (total number, percentage)	
Currently in practice	89 (93%)
Not in practice	7 (8%)
Years of practice (total number, percentage)	
0–5	17 (19%)
6–10	23 (25%)
11–15	13 (14%)
16–20	14 (15%)
21–25	8 (9%)
26–30	5 (5%)
31–35	3 (3%)
36–40	4 (4%)
41–45	4 (4%)
46–50	0 (0%)
51–55	0 (0%)
>56	0 (0%)
Additional healthcare licenses (total number, percentage)	
Advanced practice registered nurse (APRN)	11 (44%)
Registered nurse (RN)	10 (40%)
Family nurse practitioners (FNP)	2 (0.08%)
Certified hemodialysis technician (CHT)	1 (0.04%)
Clinical nurse specialist (CNS)	1 (0.04%)

* Total number, percentage.

**Table 2:** Typical procedures included in the evaluation of the temporomandibular joint (TMJ) prior to intubation.

Variable	Value *
Measurement of maximum mouth opening	64 (45%)
TMJ screening	9 (6%)
History regarding TMJ pain and/or disorders	45 (31%)
TMJ palpation	15 (11%)
Other	5 (3%)
No evaluation of TMJ health status	6 (4%)

* Total number, percentage.

**Table 3:** Reported frequency of TMJ issues and dislocation post-intubation.

Variable	Value *
TMJ issues (including pain and/or mouth limitations)	
Very frequently	0
Frequently	0
Occasionally	2 (3%)
Rarely	12 (17%)
Very rarely	29 (41%)
Never	13 (18%)
Does not evaluate	15 (21%)
TMJ dislocation	
Very frequently	0
Frequently	0
Occasionally	1 (1%)
Rarely	6 (8%)
Very rarely	38 (54%)
Never	17 (24%)
Does not evaluate	9 (13%)

* Total number, percentage.

## List of Abbreviations

TMJ	Temporomandibular joint
TMD	Temporomandibular disorders
CRNA	Certified registered nurse anesthetist
APRN	Advanced practice registered nurse
RN	Registered nurse
FNP	Family nurse practitioners
CHT	Certified hemodialysis technician
CNS	Clinical nurse specialist

## Appendix A. Survey to Anesthesia Providers

Do you agree to participate?

   Yes

   No

***Questions related to demographic and work experience:***

1. Gender
  - Male
  - Female
2. How old are you? _____________________________
3. Do you have any other healthcare licenses?
  - Yes. Please write additional licenses________________
  - No
4. Highest level of anesthesia degree completed
  - Bachelor’s degree
  - Professional/Clinical Master’s degree
  - Professional/Clinical Doctoral degree
  - Academic Master degree
  - Academic Doctoral degree (ex. PhD)
5. Are you currently in practice?
  - Yes
  - No
6. What state do you currently practice in? ______________
7. How many years have you been practicing?
  - 0–5
  - 6–10
  - 11–15
  - 16–20
  - 21–25
  - 26–30
  - 31–35
  - 36–40
  - 41–45
  - 46–50
  - 51–55
  - More than 55 years

***Questions related to general anesthesia procedures:***

8. Do you frequently perform general anesthesia with orotracheal intubation?
  - Always
  - Very Often
  - Sometimes
  - Rarely
  - Never
9. Do you consider yourself proficient regarding the performance of general anesthesia with orotracheal intubation?
  - Yes
  - Somewhat
  - No

***Questions related to temporomandibular joint (TMJ) related to anesthesia procedures:***

10. Do you think it is important to evaluate TMJ health status prior to non-emergency intubation?
  - Very Important
  - Important
  - Moderately Important
  - Slightly Important
  - Not Important
11. Do you and your team routinely evaluate TMJ health status prior to non-emergency intubation? (Including history of TMJ pain and/or disorders, current TMJ pain, mouth opening limitations, etc.)
  - Always
  - Very Often
  - Sometimes
  - Rarely
  - Never
12. What is typically included in the evaluation of the TMJ health status prior intubation? Select all that apply.
  - Measurement of maximum mouth opening
  - TMJ screening questionnaire
  - History gathering regarding TMJ pain and/or disorders
  - TMJ palpation
  - Other ____________
  - I don’t evaluate TMJ health status prior intubation.
13. Do you and your team routinely inform the patients prior to surgery about the possible risks of intubation to the TMJ?
  - Always
  - Very Often
  - Sometimes
  - Rarely
  - Never
14. In your experience, are alternative intubation equipment/techniques being selected based on the TMJ status of patients?
  - Yes
  - Sometimes
  - No
  - I don’t evaluate TMJ health status prior intubation.
15. Do you and your team evaluate TMJ health status post-intubation?
  - Always
  - Very Often
  - Sometimes
  - Rarely
  - Never
16. How frequent patients complain about TMJ issues (including pain and/or mouth opening limitations) post-intubation?
  - Very Frequently
  - Frequently
  - Occasionally
  - Rarely
  - Very Rarely
  - Never
  - I don’t evaluate TMJ health status post-intubation.
17. In your experience, how frequently do TMJ dislocation occur as a result of general anesthesia with orotracheal intubation?
  - Very Frequently
  - Frequently
  - Occasionally
  - Rarely
  - Very Rarely
  - Never
  - I don’t know
18. Are post-surgery procedures being performed related to TMJ problems after surgery?
  - Always
  - Very Often
  - Sometimes
  - Rarely
  - Never

Please write out the post-surgery procedures being performed (optional) _________

***Questions related to education on TMJ related to intubation:***

19. Did you know that tracheal intubation could cause new onset of TMJ dysfunction, or worsening of misdiagnosed TMJ disorders?
  - Yes
  - To some extend
  - No
20. Did you receive training related to TMJ issues with intubation while in school?
  - Yes
  - No
21. Do you feel that you received adequate information/education on management of TMJ issues related to intubation while in school?
  - To a Great Extent
  - Somewhat
  - Very Little
  - Not at All
22. After participating in this survey, do you think you are more aware about the importance of evaluating TMJ status before and after intubation?
  - Yes
  - No
